# Fad Diets, Body Image, Nutritional Status, and Nutritional Adequacy of Female Models in Malang City

**DOI:** 10.1155/2021/8868450

**Published:** 2021-03-11

**Authors:** Mutiara Vidianinggar, Trias. Mahmudiono, Dominikus Atmaka

**Affiliations:** Department of Nutrition, Faculty of Public Health, Universitas Airlangga, Surabaya, Indonesia

## Abstract

Female model has a variety of body image and experiences social pressure to have low weight. Thus, there is a desire to lose weight with a variety of efforts, such as fad diets. This type of diet can lead to insufficient intake of nutrients in the body and increase the risk of health problems in the long run. The research aimed to analyze fad diets, nutritional status, and nutritional adequacy of female models in Malang. The research design was cross-sectional in 52 female models aged 18–25 years who were selected by simple random sampling. The data of fad diets used in the questionnaire, nutritional status data using the calculation of the last body weight and height, and nutritional adequacy were measured using 2 × 24 hour food recall. Data analysis used the chi-square test. The results of this research showed that most respondents implement fad diets (69%), had negative body image (62%), had a skinny at high level (22%), skinny at mild level (44%), and normal (33%) of nutritional status, and had inadequate nutritional adequacy (77%). There was a significant (*p*=0.023) correlation between fad diets and nutritional adequacy (*r* = 0.369), and in addition, there was a significant correlation (*p*=0.041) between nutritional status and nutritional adequacy (*r* = 0.35). It is concluded that the female models who implemented fad diets and nutritional status below normal tended to not have adequate levels of nutritional adequacy because of improper food selection and psychological factors. Female models are expected to be able to conduct weight loss with the assistance of a nutritionist or in accordance with balanced nutrition guidelines.

## 1. Introduction

Female models often feel unsatisfied with their body shape because of social pressure that requires them to appear with strong and perfect character both technically and artistically, where their assumption is a thin body shape, low body weight, and low fat content. Demands from the agency and the environment encourage some female models to change body shape, exercise, and consumption of weight-loss pills and make efforts to lose weight by “dieting” to reach a body mass index (BMI) below normal (17.5 kg/m^2^) [1, 2]. The population of adolescents who work as models is one of the high-risk populations experiencing nutritional problems because they have a strong desire to be thinner compared to the nonmodel group [3]. Body image is the subjective imagination that a person has about his body, especially in relation to other people's judgments, and how well their body should be adjusted to these perceptions [4]. Body image is a trigger level for the emergence of eating disorders in a person [5].

The weight-loss method used by adolescent girls is on average unhealthy or commonly called fad diets [1]. The demands of the agency cause wrong dietary perspectives among the models that are caused by low knowledge and high acceptance of the sources of diet types that circulate among the people. Models believe that unhealthy eating restriction including fad diet is beneficial to their weight-loss program. The American Dietetic Association defines fad diet is diet that promises instant weight loss without having to exercise but is not based on a clear scientific basis. This type of unsafe diet can pose a risk of various health problems such as ketosis, disruption of fluid, and electrolyte balance, to cause kidney damage [6].

A diet that carried out with the wrong method continuously will result in insufficient nutrition. Inadequate nutrition can disrupt the metabolic processes in the body, and one of which is hypoglycemia. In Indonesia, there are only few research studies about eating behavior because it is still considered a trivial problem. In fact, this eating disorder can be caused by several wrong perceptions, such as the perception that a thin body is the ideal body shape, thus triggering women to make various efforts to lose weight instantly due to the low psychological aspects and knowledge aspects. Inadequate nutrient intake in the body, such as the result of fad diet, might increase the risk of health problems in the long run. Therefore, this study aimed to analyze the association between fad diets, nutritional adequacy, body image, and nutritional status of female models in Malang City. The hypothesis of this study is that fad diets can affect nutritional status and nutritional insufficiency in female models.

## 2. Materials and Methods

This research was analytical observational research with cross-sectional design. This research was conducted in March to May 20 in some model agencies such as Color Model, Posh Modeling School, Gabs Modeling, SZ Model Management, Front Row, Duta Hijab Management, and Red Modeling. The population in this research was women working as fashion models with an age range of 18–25 years. The inclusion criteria in this research were women aged 18–25 years old, registered with the agency in Malang City, active in the last 1 month, and not in a state of illness or recovering from illness while the exclusion criterion was not experiencing metabolic disorders and eating disorders. The sample size was 52 people based on the simple random sampling formula, using conventional power of 80%. Participants of this study were contacted via online in accordance to physical distancing guideline due to COVID-19 pandemic in Indonesia.

Independent variable in this research was fad diet and nutritional status while the dependent variable was nutritional adequacy. The fad diet data were obtained using measurements with the fad diet questionnaire adapted by Sulistyo [7] which has been validated for the same population characteristics (Nurjannah, 2018). This questionnaire had 10 questions consisting of questions about dietary efforts and closed questions (yes/no) about the type of fad diet that respondents have or are currently doing. The correct answer was given 2; if the wrong answer was given 1, to make the calculation easier, the question-type fad diet result was classified as doing fad diets if the score >9, and if subjects get score ≤ 9, then it is categorized as not doing fad diets. The nutritional status was obtained from measurements of body weight and height carried out during the evaluation of the agency in the past month, then included in the BMI formula from the Health Department 2013, with the category skinny at high level (BMI < 17), skinny at mild level (BMI 17.1–18.4), normal (BMI 18.5–25), mild fat (BMI 25.1−>27), and severe fat (BMI > 27.1). The body image questionnaire was adapted from the theory of Cash [8]. This questionnaire used a Likert-type scale (1 = very unsuitable; 4 = appropriate) to measure the response of the subjects. The score is obtained from the sum of all items and is categorized as a positive body image = score <115 and negative body image = score> 115. Data on the nutrition adequacy level were measured through interviews with the 2 × 24 hour food recall questionnaire for 2 nonconsecutive days. The recall results were processed average of 2 × 24 hours using NutriSurvey, and the results were compared with the 2019 Recommended Dietary Allowance (RDA) for women aged 18–25 years, which were then classified as lacking (average intake <77% RDA) or sufficient (average intake >77% RDA).

Data analysis was carried out using the statistic program through the chi-square test. The value of the chi-square test interpreted in this research was the value of *r*. It was the contingency coefficient value (*r*) which was performed to find out the strength of a relationship between variables. This study was approved by the ethics commission of the Faculty of Dentistry, Universitas Airlangga, Surabaya, on April 13^th^, 2018, under number 121/HRECC.FODM/III/2020.

## 3. Results

The results are shown in Tables 1–6.

## 4. Discussion

The results of the research showed that girl models were classified as early adulthood with an average age of 21 years. Almost all models of respondents were catagorized in early adult and had a career of 5 years and 7 months [9]. The productive period of a model started at the age of 18–25 years, or about 6 years [4]. However, there were 6 respondents or around 11.6% who had worked in the model world for more than 11 years. Their career had a large impact on their ideal weight concerns. This triggered the emergence of body dissatisfaction in female models that had an impact on weight-loss efforts with excessive dieting, which leads to eating disorders [8].

The results of this study indicate that 62% of the respondents have a negative body image. The most models of young women feel dissatisfied with their bodies because of the demands of work that require the appearance and ideal body shape [9]. A female model who has a negative body image perception will have an influence in making efforts to reduce food consumption which will lead to eating disorders. Based on Table 1, it can be seen that more than half (69.0%) of respondents currently (and ever) make efforts to lose weight by the fad diet method. The fad diet method is considered unhealthy because this diet directs a person to consume low calories and nutrients [10].

Based on Table 2, it is known that the type of fad diet that is mostly done by respondents is consuming drinks that are believed to be able to eliminate fat (slimming products such as shakers, slimming tea, and fibrous drinks) as much as 57.6% and dietary methods by consuming one type of food only (carbohydrates, protein alone, or fruit and vegetables only) with a percentage of 55.7%. It was known from the total number of teenage girls at SMAN 8, Yogyakarta, who had fad diet of 23.8% of respondents reduced certain types of food and frequency of eating [7]. Many female models had unhealthy dietary habits included skipping meals, fasting, excessive detoxification, and consumption of diet pills [2]. In addition, unequal and unequal eating habits had an impact on menstrual irregularities in women [11].

In Table 3, it is known that most respondents have underweight nutritional status. The female population who work as models is one of the high-risk populations experiencing nutritional problems because the model groups tend to be underweight [3]. Women models have a lower BMI in women in general [1]. This is quite worrying because female models who are malnourished have a greater risk of developing infectious diseases, anemia, and decreased learning achievement [5].

In Table 4, it can be seen that only 23% of respondents have nutritional adequacy according to the recommendation of the RDA, while 77% have a nutritional adequacy that is less than the RDA. This result was supported by the results of the 2010 Basic Health Research which found that 54.5% of the adolescent age population (16–18 years) had an energy intake below the minimum requirement [12].

Based on Table 5, most respondents conduct diet in wrong ways. Thus, energy intake becomes low. Respondents reduced the frequency of eating and reduced the portion of carbohydrates, not even consuming carbohydrates at all in one day. Lack of carbohydrate intake continuously will cause health problems such as chronic energy deficiency (CED), constipation, and diverticulosis [6]. The average carbohydrate intake of the female model is 161 grams (45%), whereas in reality, carbohydrate intake is important enough to maintain glycogen reserves to support the activities of the female model which are quite dense and avoid injury from rigid catwalk exercises using high wheels.

Based on Table 6, it is known that respondents who have a negative body image are more likely to have less nutritional status (93.9%), but respondents who have a positive body image also still have insufficient levels of nutritional adequacy (52.6%). In the chi-square test, the *p* value was 0.001, so it can be concluded that body image has a relationship with nutritional adequacy. And then 88.8% of respondents with a lack of adequate nutrition apply fad diets. These results indicated a dietary effort with fad diet resulted in low levels of nutritional adequacy of the respondents. The results of the analysis of the strength of the relationship showed the results of a strong correlation between fad diet and the level of nutrient adequacy. The most women who work as models have adequate levels of energy and macronutrients under the DRI (Dietary Reference Intake) and use the fad diet method by reducing the frequency of eating to 2 times a day and avoiding the consumption of carbohydrates, proteins, and fat so that they consume only fruits and vegetables [13].

Fad diets are a common method used by people with rapid weight loss, but only the amount of water and muscle loss, not fat tissue [14]. Other fat diet methods such as low-carbohydrate/high-protein and fat diets cause a change in the main energy source, from glucose to fatty acids and ketones, which can cause ketosis. Ketosis has been shown to reduce weight and improve body fat and glucose profiles, but this diet has both acute and chronic risks [15]. Carbohydrate consumption can affect human cognitive function, because the brain consumes the most energy from glucose, which is 20% of the total energy intake that enters the body [16].

This research has point of combining elements of psychology with nutritional aspects which are felt to affect the health problems of female models in the long run. However, this research has limitations, namely, interviews and questionnaires are conducted online (Google form and video call), since the time of the study coincided with the COVID-19 pandemic disaster and the existence of a social distancing policy which resulted in the agency not scheduling training and limiting meetings with respondents. Researchers cannot control for other factors that can confound and influence respondents, such as when conducting recall interviews between stays home describing eating patterns that may be different from daily eating patterns, thus allowing for bias.

## 5. Conclusions

Most women have negative body image and nutritional status below normal (underweight) with the category of mild-to-thin underweight. Some respondents who have negative body image and nutritional status below normal are currently (or ever) applying the fad diets diet method by consuming drinks that are believed to eliminate fat (slimming products such as shakers, slimming teas, and fibrous drinks), consuming only one type of food (carbohydrates only, protein only, or fruit and vegetables only), resulting in inadequate nutritional intake in the body. The female model is expected to increase the frequency of eating in a day, which was originally from twice a day to three times a day by implementing a balanced nutritionally appropriate diet through consultation with a nutritionist to achieve an ideal body.

## Figures and Tables

**Table 1:** The distribution of respondents' characteristic, fad diets, and body image of female models in Malang City.

Characteristic of respondent	Number (*n*)	Percentage (%)
Age (year)		
18–20	18	35
21–23	21	40
23–25	13	25

TB (cm)		
156–161	12	23
162–167	15	29
168–173	19	37
174–179	6	11

BB (kg)		
40–45	10	19
46–50	21	40
51–55	14	27
56–60	7	14

Type fad diets		
Fad diets	36	69
Not fad diets	16	31

Body image		
Negative	33	62
Positive	19	38

**Table 2:** The distribution of fad diets based on the questionnaire.

Question of body image questionnaire	*n*	(%)
Diet method I apply is decreasing eating frequency in a day (such as skipping breakfast/dinner)	26	50
Diet method I apply is eating certain combinations of food (for example, rice, vegetables, and protein during the day, rice and vegetables at night, fruit only on the first day, etc.)	17	32.6
Diet method I apply is avoiding certain foods (carbohydrates, sweet foods, and fatty foods) and replacing them with vitamin and mineral supplements	24	46.1
Diet method I apply is consuming just one particular type of food (for example, just carbohydrates, only protein, only fruits, and vegetables)	29	55.7
Diet method I apply is consuming drinks that are believed to eliminate fat (for example, slimming products such as shakes, slimming tea, and fibrous drinks)	30	57.6
Diet method I apply is taking diet pills (slimming pills and fat removal pills)	26	50

**Table 3:** The distribution of nutritional status of female models in Malang City.

Category	Number (*n*)	Percentage (%)
Skinny at high level	12	23
Skinny at mild level	23	44
Normal	17	33

**Table 4:** The distribution of nutritional adequacy of female models in Malang City.

Category	Number (*n*)	Percentage (%)
Adequate	12	23
Not adequate	40	77

**Table 5:** The distribution of respondents based on level of energy adequacy and macronutrition of female models in Malang City.

Nutrient	Adequate		Less	
	*n*	(%)	*n*	(%)
Energy (kcal)	9	17.3	43	82.7
Carbohydrate (g)	5	9.6	47	90.4
Protein (g)	29	55.7	23	44.3
Fat (g)	27	51.9	25	48.1

**Table 6:** The relation of fad diets, body image, nutritional status, and adequacy of nutrition of female models.

Variable	Adequacy of nutrition						*p* value	*r* value
	Less		Adequate		Total			
	*n*	(%)	*n*	(%)	*n*	(%)		
Fad diets								
Yes	32	88.8	4	11.2	36	100	0.023	0.369
No	9	56.25	7	43.75	16	100		

Body image								
Negative	31	93.9	2	6.1	33	100	0.001	0.487
Positive	10	52.6	9	47.4	19	100		

Nutritional status								
Skinny at mild level	21	91.3	2	0.7	23	100	0.041	0.350
Skinny at high level	10	83	2	17	12	100		
Normal	10	58.8	7	41.2	17	100		

## Data Availability

The data used to support the findings of this study are available from the corresponding author upon request.
